# Pathogenic Homocystinuria-Associated T236N Mutation Dramatically Alters the Biochemical Properties of Cystathionine Beta-Synthase Protein

**DOI:** 10.3390/biomedicines12050929

**Published:** 2024-04-23

**Authors:** Duaa W. Al-Sadeq, Angelos Thanassoulas, Maria Theodoridou, Gheyath K. Nasrallah, Michail Nomikos

**Affiliations:** 1 Biomedical Research Center, Qatar University, Doha P.O. Box 2713, Qatar; da1206066@qu.edu.qa (D.W.A.-S.); gheyath.nasrallah@qu.edu.qa (G.K.N.); 2 College of Medicine, QU Health, Qatar University, Doha P.O. Box 2713, Qatar; angelosthanassoulas@gmail.com; 3 Department of Biomedical Science, College of Health Sciences, QU Health, Qatar University, Doha P.O. Box 2713, Qatar

**Keywords:** homocystinuria, cystathionine beta-synthase, pyridoxine non-responsiveness, mutation, circular dichroism

## Abstract

Background: Cystathione beta-synthase (CBS) T236N is a novel mutation associated with pyridoxine non-responsiveness, which presents a significant difficulty in the medical treatment of homocystinuria. Reported severe phenotypes in homocystinuria patients highlight the urgent requirement to comprehend the molecular mechanisms underlying mutation pathogenicity for the advancement of the disease. Methodology: In this study, we used a multidisciplinary approach to investigate the molecular properties of bacterially expressed and purified recombinant CBS^T236N^ protein, which we directly compared to those of the wild-type (CBS^WT^) protein. Results: Our data revealed a profound impact of the p.T236N mutation on CBS enzymatic activity, with a dramatic reduction of ~96% compared to the CBS^WT^ protein. Circular dichroism (CD) experiments indicated that the p.T236N mutation did not significantly alter the secondary structure of the protein. However, CD spectra unveiled distinct differences in the thermal stability of CBS^WT^ and CBS^T236N^ mutant protein species. In addition, chemical denaturation experiments further highlighted that the CBS^WT^ protein exhibited greater thermodynamic stability than the CBS^T236N^ mutant, suggesting a destabilizing effect of this mutation. Conclusions: Our findings provide an explanation of the pathogenicity of the p.T236N mutation, shedding light on its role in severe homocystinuria phenotypes. This study contributes to a deeper understanding of CBS deficiency and may improve the development of targeted therapeutic strategies for affected individuals.

## 1. Introduction

CBS deficiency represents a major challenge in the field of hereditary metabolic disorders and is often manifested by homocystinuria, a condition characterized by elevated homocysteine levels in the blood and urine [1]. Elevated levels of homocysteine can lead to various complications and are associated with several pathological disorders. It is considered a risk factor for conditions such as cardiovascular diseases, neurodegenerative diseases, renal dysfunction, cognitive impairments, and congenital defects [2,3]. The presence of homocysteine in excess amounts can impact bone health, contribute to vascular diseases like coronary heart disease, and increase the risk of acute events like stroke. Furthermore, homocysteine metabolism disruptions due to factors like vitamin B deficiencies or genetic defects can result in the accumulation of homocysteine, emphasizing the importance of monitoring and managing homocysteine levels to prevent associated health complications [4]. Homocystinuria poses a significant burden of disease globally, with varying frequencies observed across different countries. The prevalence of homocystinuria exhibits significant variation, with estimated rates ranging from 1 in 200,000 to 1 in 350,000 live births globally, but may be as low as 1 in 1,000,000 in some populations [5,6]. However, certain populations exhibit a notably higher frequency of CBS deficiency, underscoring the importance of geographical and ethnic factors in disease prevalence. For instance, Qatar has been reported to have the highest frequency of CBS deficiency compared to other countries (1:1800) [7,8]. This is due to the homozygous c.1006C>T (p.R336C) mutation found in *CBS* gene exon 9, leading to severe vitamin B6 non-responsive phenotypes. This mutation’s prevalence in Qatar highlights the challenges faced in managing homocystinuria due to its pyridoxine non-responsive nature and strict dietary requirements. The burden of homocystinuria in Qatar necessitates urgent attention to develop tailored treatment strategies and enhance clinical outcomes within the population. Despite significant advances, the intricacies of CBS deficiency remain complex, with a notable subset of patients exhibiting an intriguing phenomenon known as pyridoxine responsiveness [9]. They are subdivided into three main categories, partial, full, and extreme responders to pyridoxine. This feature, observed in approximately 30–50% of cases, highlights the diverse clinical manifestations within the CBS deficiency spectrum. However, within this diverse landscape, a distinct cohort is emerging: those who demonstrate pyridoxine non-responsiveness. This subgroup represents a unique conundrum and requires a deeper exploration of the underlying molecular mechanisms, to enable more targeted therapeutic approaches.

The *CBS* gene encodes a crucial enzyme involved in sulfur amino acid metabolism, catalyzing the condensation of serine and homocysteine to form cystathionine [10]. This enzyme plays a vital role in the transsulfuration pathway, contributing to the biosynthesis of cysteine and the regulation of homocysteine levels. The CBS protein consists of several functional domains essential for its enzymatic activity and regulation. At its core, the protein contains a pyridoxal-5′-phosphate (PLP)-binding domain, crucial for its catalytic function in the condensation of serine and homocysteine. Adjacent to the PLP-binding domain lies the CBS domain, named after cystathionine beta-synthase, which plays a regulatory role in the enzyme’s activity. Additionally, the CBS protein possesses a heme-binding domain, enabling it to function as a redox sensor and modulate enzyme activity in response to changes in cellular redox status. These distinct domains within the CBS protein contribute to its multifaceted roles in sulfur amino acid metabolism and underscore its significance in cellular homeostasis [11]. Understanding the structure and function of the CBS protein is essential for elucidating the pathophysiology of CBS deficiency and developing targeted therapeutic interventions.

To date, a diverse array of mutations has been identified in the *CBS* gene, contributing to the pathogenesis of CBS deficiency. These mutations encompass various types, including missense, nonsense, frameshift, and splicing mutations, highlighting the genetic heterogeneity of this disorder.

A missense mutation in the *CBS* gene p.T236N was identified in individuals exhibiting pyridoxine non-responsiveness. This mutation is characterized by the substitution of threonine (T) with asparagine (N) at position 236 within the CBS protein sequence and is located in the protein catalytic domain. This domain plays a pivotal role in coordinating interactions with cofactors and modulating enzymatic function. The substitution at position 236 may disrupt critical interactions within the CBS domain, thereby impairing the protein’s catalytic activity and leading to the clinical manifestations associated with CBS deficiency. Understanding the precise nature and location of the p.T236N mutation within the *CBS* gene and protein domain is essential for elucidating its impact on protein structure and function, thereby improving strategies for diagnosis, management, and therapeutic intervention in individuals affected by CBS deficiency. To gain a better understanding of the molecular mechanism of this *CBS* mutation, which leads to the pathogenic phenotype, we initially used a bacterial expression system to generate the human CBS^WT^ and its corresponding CBS^T236N^ mutant as recombinant proteins with the aim to directly compare their biochemical and biophysical properties. We used a multidisciplinary approach including enzymatic assays, circular dichroism, chemical denaturation experiments and molecular simulations. Our study is the first to report the dramatic consequences of the T236N mutation on the stability and enzymatic function of CBS protein, contributing to a better understanding of the complex mechanisms of those certain *CBS* mutations that lead to homocystinuria pyridoxine non-responsiveness. 

## 2. Materials and Methods 

### 2.1. Generation of Constructs

Human *CBS* (NCBI reference sequence NM_000071.2) was amplified by PCR from pLW2:hCBS. CBS^WT^ and CBS^T236N^ mutants were then cloned in the pET28a vector between NdeI and XhoI restriction sites. The primers used for the amplification of the CBS constructs were 5′-CAGTCATATGCCTTCTGAGACCCCCCA-3′ (forward) and 5′-GATACTCGAGTCACTTCTGGTCCCGCTC-3′ (reverse).

### 2.2. Recombinant Protein Expression and Purification

Affinity chromatography serves as a crucial method for selectively isolating and purifying enzymes, leveraging their unique ability to bind ligands specifically and reversibly. In this study, human CBS^WT^ and CBS^T236N^ constructs were transformed in competent *E. coli* [BL21-CodonPlus (DE3)-RILP cells (Stratagene, La Jolla, CA, USA). Cells were cultured at 37 °C until the optical density at 600 nm reached 0.6. Recombinant CBS proteins were then extracted and purified. The recombinant proteins were engineered with a 6xHis tag at the N-terminus, enabling their purification via immobilized metal affinity chromatography (IMAC) utilizing a Ni^2+^-chelating resin [QIAGEN (Hilden, Germany). The process involved suspending a pellet of *E. coli* BL21 (DE3) cells in lysis buffer, followed by ultra-sonication and centrifugation to obtain insoluble (pellet) and soluble (supernatant) fractions. After preliminary characterization using Sodium Dodecyl Sulphate–Polyacrylamide Gel Electrophoresis (SDS-PAGE), the supernatant was loaded into a Ni-NTA column, and various wash buffers were applied to remove impurities. An elution buffer was then used to release the recombinant proteins, which were subsequently dialyzed, and the protein concentration was estimated using the BCA assay. Samples were concentrated as needed using ultrafiltration and stored at −80 °C for future use. This process underscores a meticulous approach to protein purification, ensuring the quality and integrity of the obtained proteins.

### 2.3. SDS Polyacrylamide Gel Electrophoresis and Western Blotting

By combining the purified proteins with SDS, which disperses them evenly by binding to hydrophobic regions and causing them to unfold, larger proteins separate from smaller ones during electrophoresis. The process includes a stacking gel and a resolving gel, with percentages adjusted based on protein size. In this study, a 12% resolving gel and a 4% stacking gel were prepared, catalyzed by APS and TEMED. Denatured cell lysates were loaded after heating and electrophoresis was conducted in running buffer, followed by visualization using a Brilliant Blue Coomassie stain with PageRuler™ Prestained Protein Ladder (Thermo Scientific, Vilnius, Lithuania) for molecular weight estimation. After conducting SDS-PAGE electrophoresis to separate the proteins, we transferred them onto a polyvinylidene difluoride (PVDF) transfer membrane (Thermo Scientific, Rockford, IL, USA) using a semi-dry transfer system. Following a one-hour transfer at 20 V, we confirmed the efficacy using Ponceau S stain. The membrane was then blocked with 5% skimmed milk in TBST buffer before incubating with anti-CBS mouse polyclonal antibody overnight at 4 °C. The next day, the membrane was probed with IgG goat anti-mouse secondary antibody conjugated with HRP for one hour at room temperature. After washing, we developed the immunoblot using the ECL Western Blotting Substrate Kit (Thermo Scientific, Rockford, IL, USA) and captured the luminescence signal with the Invitrogen iBright CL1000 imaging system (Thermo Scientific, Carlsbad, CA, USA). Quantification of protein bands was performed using ImageJ software version 1.54.

### 2.4. Enzymatic Assays

The enzymatic activity of the purified recombinant CBS^WT^ and CBS^T236N^ proteins was assessed in a final reaction volume of 200 μL, following previously outlined methods with slight adjustments [12,13]. The reaction mixture included 30 μL of varying protein concentrations (ranging from 0.1 µg to 5 µg) combined with 170 μL of master mix. Fluorescence readings were promptly recorded at excitation/emission wavelengths of 368/460 nm in kinetic mode over a duration of 40–60 min at 37 °C. The specific enzyme activity was quantified as U/mL, representing the quantity of the enzyme required to produce 1 nmol of 7-amino-4-methylcoumarin per minute under standardized conditions of pH 8.0 at 37 °C.

### 2.5. Circular Dichroism and Thermal Denaturation Experiments

Circular dichroism (CD) experiments were conducted utilizing a J-1100 spectropolarimeter (JASCO, Tokyo, Japan) equipped with a Peltier-type cell holder for precise temperature regulation. Spectral scans ranging from 195 to 260 nm in the far-UV region were collected employing Quartz SUPRASIL (HELLMA) precision cells with a path length of 0.1 cm. The cuvette was filled with 250 μL of a 5 μΜ protein solution in PBS buffer for these measurements. The final spectrum involved the averaging of eight consecutive accumulations, utilizing a wavelength increment of 0.2 nm at a velocity of 20 nm min^−1^, a response time of 1 s, and a bandwidth of 1 nm. Subtraction of the buffer spectra from the sample scans was performed accordingly, using identical settings. As a final step, the data from all scans were normalized as molar ellipticity [θ] (units: deg cm^2^ dmol^−1^).

In the thermal denaturation measurements, the CD signal was observed at 212 nm as the sample’s temperature gradually rose from 25 to 90 °C in steps of 0.5 °C and a heating rate of 1.5 °C/min. The sample used for each melting experiment was 250 μL of a 5 μM protein solution in PBS buffer, loaded into a 0.1 cm path length Quartz SUPRASIL (HELLMA) precision cell. The subtraction of buffer blank spectra, acquired under the same conditions, was conducted on the initial data. The outcomes of all heat-induced unfolding studies were normalized according to the method described by Greenfield [14] and plotted as the proportion of the unfolded population relative to temperature.

### 2.6. Chemical Denaturation Experiments

Fluorescence measurements were conducted using a Horiba Fluoromax 4 spectrofluorometer (HORIBA Advanced Techno—Kyoto, Japan) equipped with a xenon short-arc lamp (Ushio) and a 4 mL quartz cuvette (Roth, Germany). The denaturation profiles of recombinant CBS proteins were monitored by incrementally introducing small volumes of an 8 M guanidinium chloride (GuHCl) solution into a cuvette with a 1 cm path length, containing 0.05 mg/mL of the protein in PBS buffer. Subsequent to each injection, the solution was mixed and left to equilibrate at 25 °C for 15 min. Emission spectra ranging from 300 to 450 nm were collected after excitation at 280 nm. Emission signals from the buffer solution at varying GuHCl concentrations were obtained separately and subtracted from the final dataset. To maintain a constant protein concentration, an appropriate quantity of a concentrated protein solution was added to the cuvette prior to each reading. The titration process was stopped when a 4.5 M GuHCl concentration was reached in the sample. The denaturation data were analyzed using a two-state model that elucidates the induced unfolding through a single transition between the native (N) and unfolded (U) states:

$$F=\frac{\left(\alpha_{Ν}+\beta_{Ν}\left[D\right]\right)+\left(\alpha_{U}+\beta_{U}\left[D\right]\right){\cdot e}^{\left(\frac{m\left(\left[D\right]-{\left[D\right]}_{50\%}\right)}{RT}\right)}}{1+e^{\left(\frac{m\left(\left[D\right]-{\left[D\right]}_{50\%}\right)}{RT}\right)}}$$

The weighted average emission wavelength, denoted by F, is determined in relation to the concentration of the denaturant in the sample, represented as [D], alongside the signal of the native state at 0 M denaturant concentration, *α_N_*, the signal slope at the native state denoted by *β_N_*, and the corresponding quantities for the unfolded state, *α_U_* and *β_U_*. The proportionality constant, m, is defined as the derivative of the free-energy change between the native and unfolded states with respect to the denaturant concentration, and [D]_50%_ signifies the denaturant concentration at which the protein population is 50% unfolded. By employing nonlinear least-squares fitting methods to solve the equation, values for [D]_50%_ and m can be obtained along with their standard deviations. The calculation of the free-energy change from the native to unfolded states is then determined as ΔG_U-N_ = m·[D]_50%_.

### 2.7. Molecular Simulations

The GROMACS 5.18.3 software package was utilized for all molecular dynamics simulations [15,16]. The molecular dynamics (MD) simulations were based on structural templates derived from the models predicted by Alphafold for the monomeric human CBS protein (Entry P35520) [17]. Utilizing the GROMOS 54A7 force field, topology parameter files for both WT and T236N were generated [18]. Incorporation of a single point charge water model (SPC) facilitated the solvation of all protein models in the investigation, positioning the protein at the center of a cubic grid box (total volume: 1 nm^3^) [19]. 

To achieve system neutrality, NaCl was introduced to reach a final molarity of 0.15 M. The ionization states of the residues were determined at pH 7, assuming a neutral deprotonated state for all histidines. Following system neutralization, an energy minimization procedure was implemented utilizing the Steepest Descent (SD) method for 5000 iterations, succeeded by two equilibration periods—first a 500 ps duration in an NVT ensemble, and subsequently a 500 ps period in an NPT ensemble. The pressure and temperature of the system were held constant at 1 bar and 300 K, respectively, using a coupling time constant of 1.0 ps. A cut-off of 1.0 nm was applied to all van der Waals and Coulomb interactions within the simulation box. Subsequently, MD simulations were carried out on all protein systems for a duration of 10 ns, with trajectory data being gathered at intervals of 200 ps. The selection of representative structures for this research was conducted by clustering the MD simulation trajectories with the help of the gmx cluster analysis tool within GROMACS, following the Gromos method [20]. The root-mean-square deviation (RMSD) and root-mean-square fluctuation (RMSF) were calculated from the trajectory files utilizing the g_rmsd and g_rmsf tools in GROMACS. The determination of the radius of gyration (k) and the solvent-accessible surface area (SASA) at each simulation step was achieved by using the g_gyrate and g_sas commands, respectively.

## 3. Results and Discussion

The present study investigated a novel homozygous mutation, c.707C>A (T236N), in a Chinese patient with homocystinuria. The mutation is located in exon 8 and was reported to be novel. The patient was diagnosed with classical homocystinuria at the age of 11 [15]. The major clinical features of the patient included skeletal abnormality, developmental delays, and eye disorders. More specifically, the patient showed osteoporosis and eye disorders, including ectopia lentis. Significantly increased plasma (222 μmol/L) and urinary (<500 μmol/L) total homocysteine were observed. The patient’s blood methionine levels elevated to 468 μmol/L (normal range 10–50 μmol/L). Similar clinical features and elevated homocysteine were reported in a Han Chinese family with two novel *CBS* mutations [21]. Interestingly, the patient was unresponsive to vitamin B6 (a PLP precursor) supplementation, as reported in the clinical data [22]. This pathogenic association prompted us to evaluate the biochemical phenotypes associated with the variant. Therefore, we aimed to evaluate the enzymatic activity of the human CBS^T236N^ mutant at different concentrations with the addition of pyridoxal and heme cofactors and compare it directly with the enzymatic activity of the CBS^WT^ protein.

**Figure 1:**
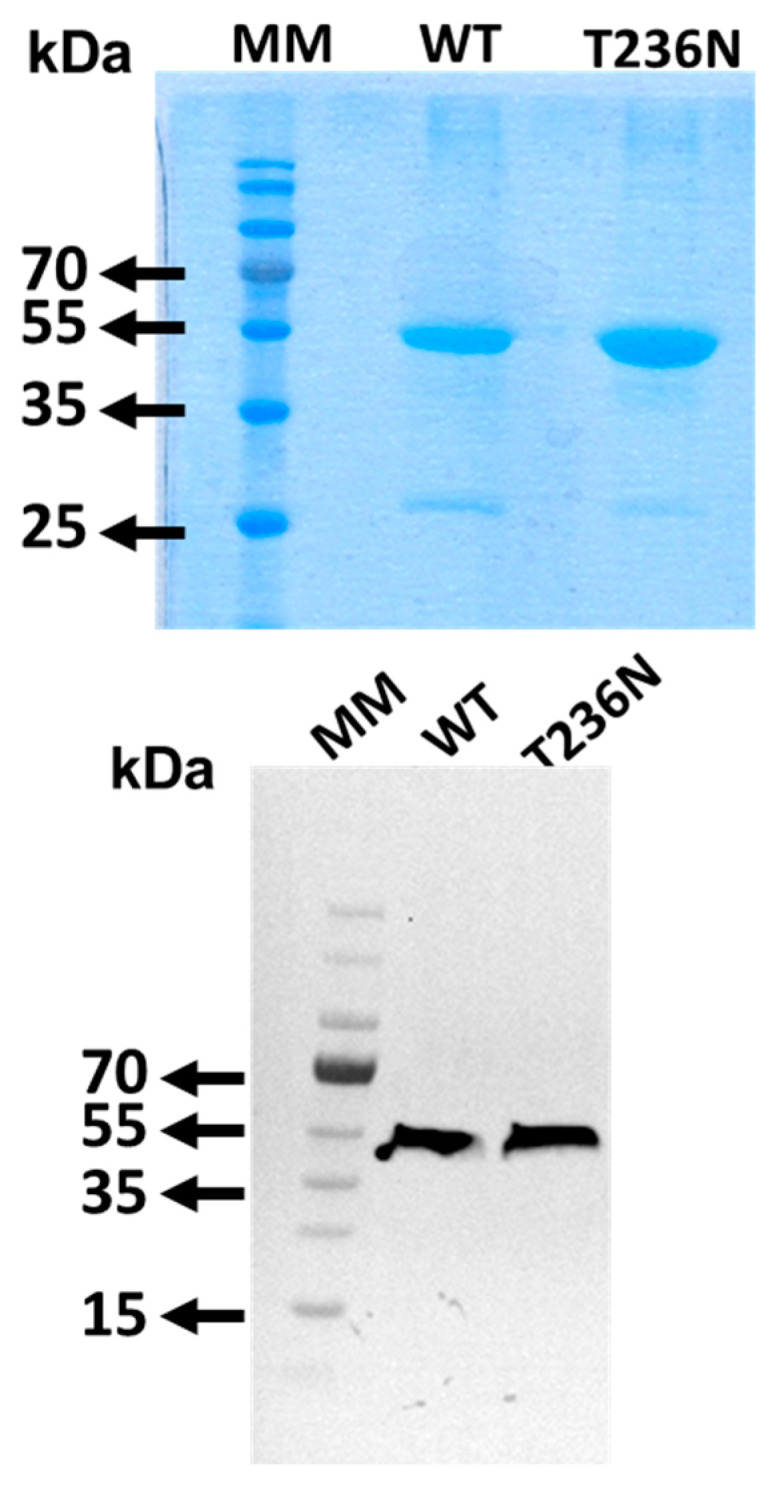
10% SDS-PAGE analysis of purified recombinant CBS variants by Coomassie Brilliant Blue (**upped panel**) and Western blotting (**lower panel**) with mouse anti-CBS polyclonal antibody (Abnova) at 1:1000 dilution.

CBS^WT^ and CBS^T236N^ mutant proteins were expressed using a bacterial expression system and purified to homogeneity as we have previously described [13]. To confirm the identity of the recombinant CBS proteins, SDS-PAGE analysis followed by Coomassie Brilliant Blue and Western blotting using an anti-CBS antibody was performed (Figure 1).

**Figure 2:**
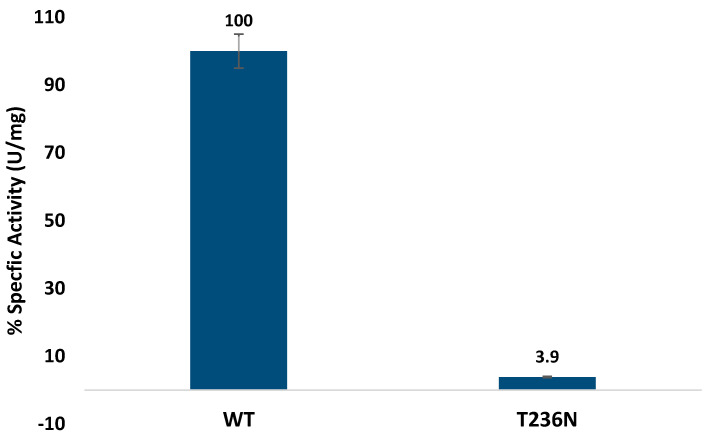
CBS^WT^ and CBS^T236N^ enzymatic activity.

Activity was determined by measuring fluorescence emission at 460 nm (excitation at 368 nm) [12]. Our enzymatic assays demonstrated a drastic reduction in CBS activity upon introduction of the p.T236N mutation, consistent with its classification as a pyridoxine non-responsive mutation (Figure 2). This severe impairment in enzymatic function was similar to previous reports of other *CBS* mutations in the catalytic domain [10,23]. However, our study is the first to characterize and assess the CBS^T236N^ mutation activity. The observed reduction in enzymatic activity highlights the importance of pyridoxal and heme cofactors in modulating CBS function, further emphasizing the potential therapeutic implications of cofactor supplementation in certain CBS deficiency cases [24]. Our results showed similar impaired activity for other *CBS* missense mutations with prior investigations that evaluated enzymatic activity using diverse methodologies. For instance, comparable enzymatic profiles were noted in a prior analysis of 14 mutations within the human *CBS* gene aimed at assessing their potential pathogenicity. Of these mutations, eleven exhibited activity levels below 4% of the CBS^WT^ protein, reinforcing the pathogenic nature of these mutations [25].

**Figure 3:**
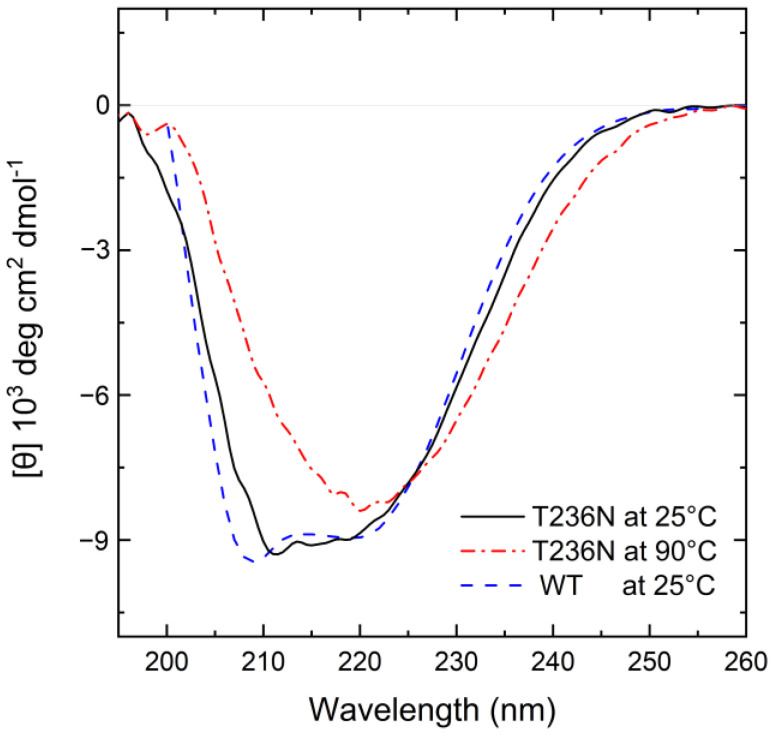
Far-UV CD spectra of 5 μΜ CBS^WT^ and CBS^T236N^ proteins in PBS buffer. The black solid line corresponds to CBS^T236N^ at 25 °C, the red dash-dotted line corresponds to CBS^T236N^ at 90 °C, while the blue dashed line corresponds to CBS^WT^ at 25 °C for comparison purposes.

The far-UV spectrum of CBS^T236N^ at 25 °C revealed minor structural alterations associated with the p.T236N mutation at the same temperature (Figure 3). Analysis of the spectra using the BESTSEL online server [26] shows that the CBS^T236N^ mutant has a lower antiparallel (12.9%) and a higher parallel (14.3%) β-sheet content when compared to CBS^WT^ (19.9% and 6.1%, respectively), indicating some differences in the overall protein architecture (Table 1). Heating CBS^T236N^ to 90 °C only partially unfolds the structure, with the protein retaining a significant portion of its secondary structure elements. However, this thermal transition is irreversible since no further changes in the far-UV CD spectrum were observed when the sample was cooled to 25 °C and visible protein aggregates formed in the cuvette as a direct result of the heating step.

**Figure 4:**
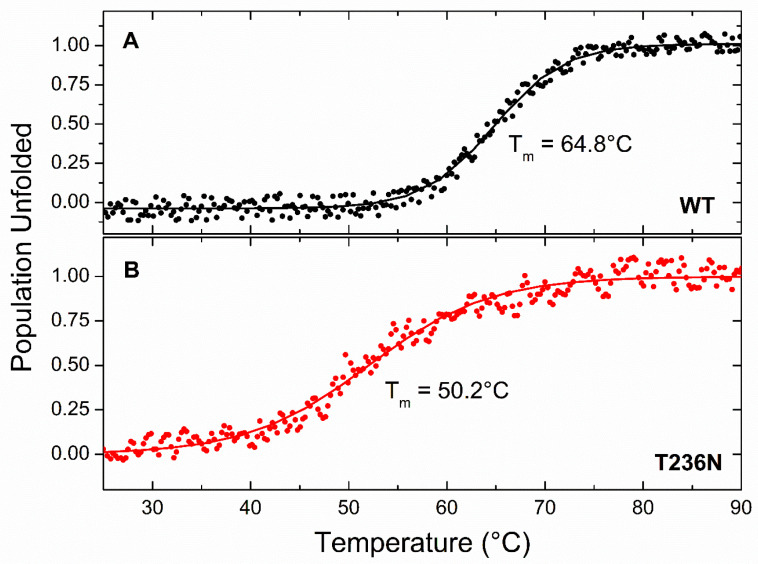
Thermal denaturation profiles of 5 μΜ CBS^WT^ (**A**) and CBS^T236N^ (**B**). CBS proteins in PBS buffer, monitored at 212 nm and normalized as the unfolded protein population. Circles represent experimental data and solid lines correspond to sigmoid fits as a guide to the eye.

The thermal stability of CBS^WT^ and CBS^T236N^ was examined by heating the proteins at a steady rate from 25 °C to 90 °C and monitoring CD changes at 212 nm. The raw data were normalized as an unfolded protein population for comparison purposes, and the results are shown in Figure 4. Both the CBS^WT^ and CBS^T236N^ proteins unfold following a simple two-state transition, as shown by the characteristic sigmoidal melting curves. The CBS^T236N^ unfolding process exhibits lower comparativity compared to that of CBS^WT^, as indicated by the temperature change required (ΔΤ) for a fully folded protein population to become unfolded (ΔT^T236N^ ~ 40 °C and ΔT^WT^ ~ 25 °C, respectively). Interestingly, CBS^T236N^ appears to be significantly thermally destabilized, as the melting temperature of the transition (T_m_, temperature at which only 50% of the protein population is folded) is almost 15 °C lower than that of the CBS^WT^ (T_m_^T236N^ = 50.2 °C and T_m_^WT^ = 64.8 °C, respectively). However, the non-reversibility of the transitions does not allow for a more detailed thermodynamic analysis of these data. Threonine’s hydroxyl group can participate in hydrogen bonding interactions, contributing to protein stability and folding [27]. Asparagine’s amide group can also engage in hydrogen bonding, albeit differently from the hydroxyl group. The substitution may affect the network of hydrogen bonds within the protein, influencing its stability and function.

**Figure 5:**
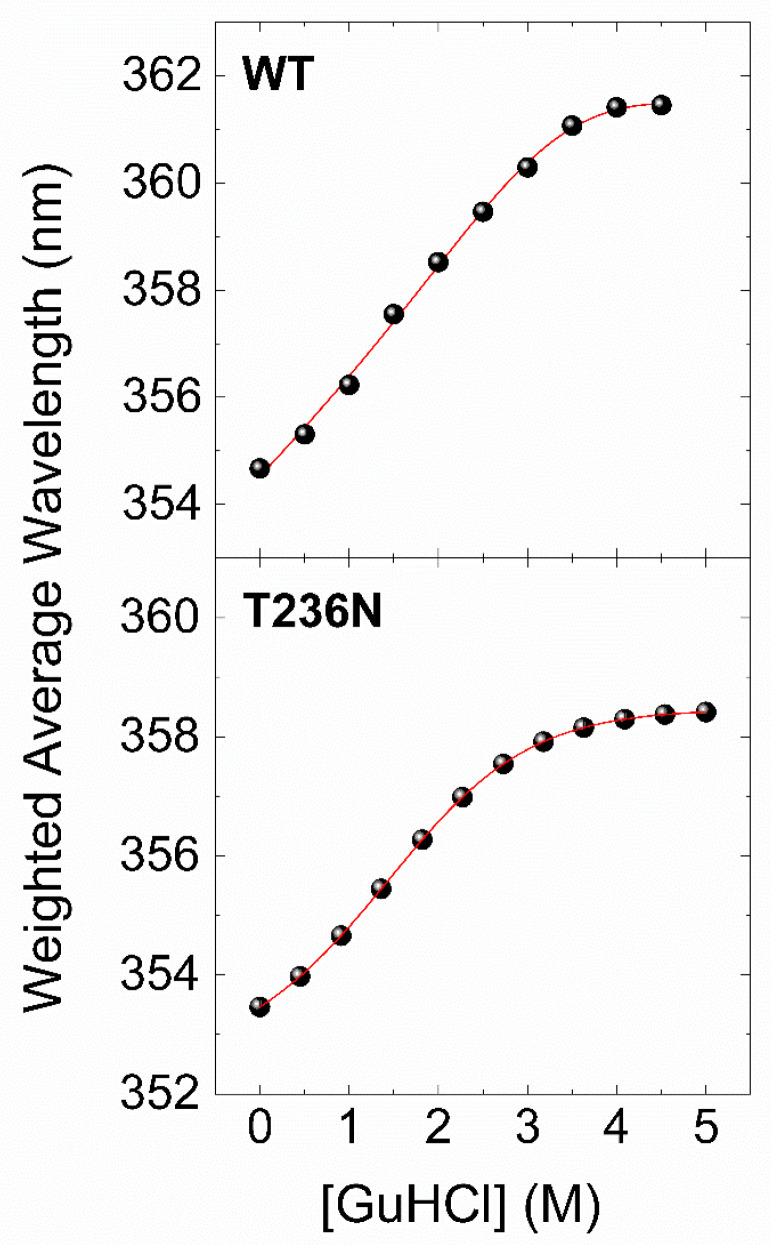
Chemical denaturation profiles of 0.05 mg/mL CBS^WT^ (**upper panel**) and CBS^T236N^ (**lower panel**) proteins in PBS buffer at 25 °C, plotted as weighted average emission wavelengths at various denaturant concentrations. Solid lines correspond to nonlinear least-squares fits of a simple two-state thermodynamic model with the experimental data.

To further investigate the impact of p.T236N on protein stability, we used chemical denaturation assays to compare recombinant CBS^WT^ and CBS^T236N^ proteins. This was achieved by monitoring changes in the fluorescence emission spectrum of the protein in the 300–450 nm range at different concentrations of a chemical denaturant. Guanidinium chloride (GuHCl) was used as a denaturant due to its ability to reversibly weaken or break noncovalent interactions, including salt bridges and hydrogen bonds [28]. The data of the chemical denaturation assays were processed as described in the Section 2, and the final results are presented in Figure 5 as plots of the weighted average emission wavelength versus GuHCl concentration. Similar to the thermal transitions of CBS^WT^ and CBS^T236N^, the chemical denaturation profiles show a two-state unfolding process for both proteins, with the fluorescence emission spectra shifting towards higher wavelengths as the concentration of the denaturant increases. However, in this case, the reversibility of the process (dialysis of the samples back to the original buffer conditions gives almost identical emission spectra) allows for a more in-depth thermodynamic analysis. Table 2 summarizes the thermodynamic stability parameters derived by a nonlinear least-squares curve fitting of a simple two-state transition model with our experimental data (Figure 4, solid lines). The results showed that the free-energy change difference (ΔΔG_N-U_) between CBS^WT^ and CBS^T236N^ was 1.26 kcal/mol, indicating that the CBS^WT^ protein is thermodynamically more stable than the CBS^T236N^ mutant. Taken together, CD and fluorescence data strongly suggest that CBS^T236N^ has a destabilizing effect on the CBS protein.

**Figure 6:**
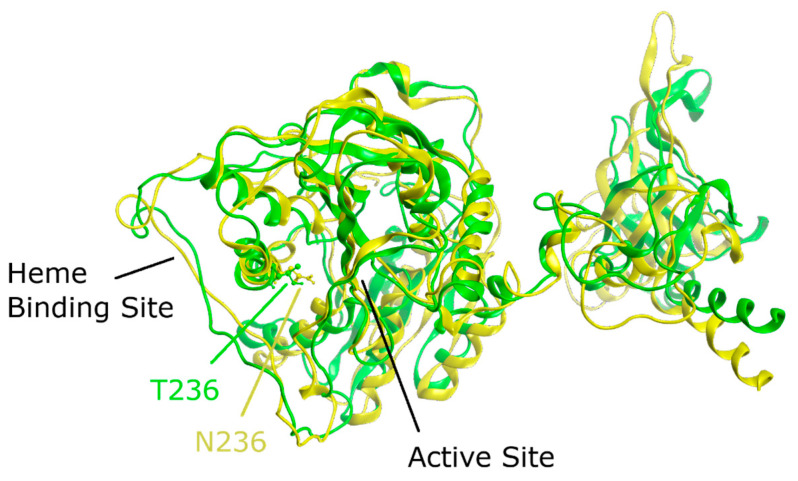
Overlayed representative MD structures for CBS^WT^ (green ribbons) and CBS^T236N^ (yellow ribbons). Residues at position 236 are shown as ball–stick models, while the black lines show the location of the cofactor binding sites.

**Figure 7:**
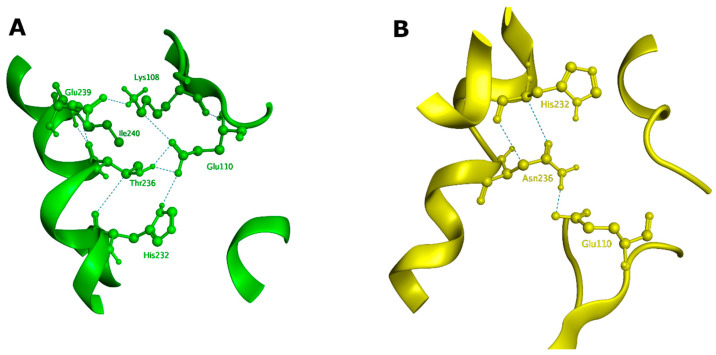
Local interactions at position 236 for CBS^WT^ (**A**) and CBS^T236N^ (**B**) based on the representative structures of the MD simulations. In the case of CBS^T236N^, only the His232 and Glu110 interactions are preserved from the original network of stabilizing bonds. All interactions shown in this figure (green dashed lines) represent hydrogen bonds with energies of at least −1 kcal/mol.

**Figure 8:**
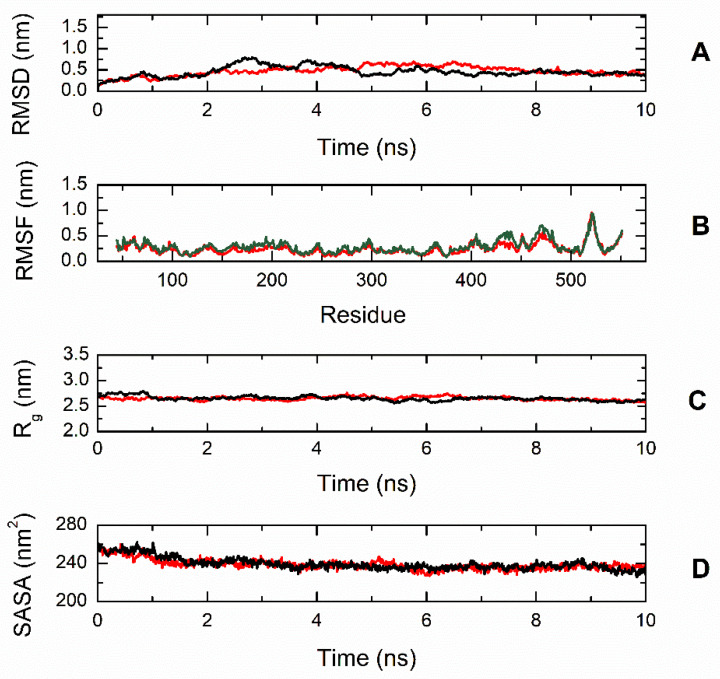
Trajectory analysis of CBS^WT^ (black, ―) and CBS^T236N^ (red, ―) proteins. (**A**) Root-mean-square deviation (RMSD) of the protein backbone atoms as a function of MD simulation time. (**B**) Root-mean-square fluctuations (RMSFs) of individual residues for the duration of the simulation. (**C**) Total radius of gyration (R_g_) of the protein backbone atoms as a function of MD simulation time. (**D**) Solvent-accessible surface area (SASA) of the protein as a function of MD simulation time. All parameters indicate a similar dynamic behavior for WT and mutant CBS.

To gain better insight into the effect of the mutation on protein structure and dynamics, we performed a series of molecular dynamics simulations for both CBS^WT^ and CBS^T236N^ proteins. An overlay of the representative structural models for CBS^WT^ and CBS^T236N^ is shown in Figure 6. The MD results suggest that both proteins adopt a similar conformation; however, small changes in secondary structure elements and in their relative position are evident throughout the protein structure, consistent with our far-UV CD data (root-mean-square deviation (RMSD) = 4.4 Å). Analysis of the local interactions for T236 in WT and N236 in p.T236N (Figure 7) shows that the network of stabilizing bonds is significantly less extensive in the case of the mutant, which may justify the reduced stability (both thermal and thermodynamic) observed during our CD and fluorescence experiments. Further analysis of the simulation trajectories revealed almost identical behavior for both proteins in terms of RMSD, root-mean-square fluctuations (RMSFs), radius of gyration (R_g_) and solvent-accessible surface area (SASA) over the simulation time, suggesting a minimal impact of the mutation on the protein dynamics and general folding (Figure 8).

Despite the extensive research conducted over the past three decades, there remains a scarcity of comprehensive molecular and biochemical characterizations for CBS missense mutations. These pathogenic mutations have the potential to profoundly impact the structural and functional integrity of proteins. Regarding CBS, more than 200 patient mutations have been documented to date, distributed across all three domains of this modular protein [29]. Our study represents the first comprehensive characterization of the novel CBS p.T236N mutation. By employing a range of biochemical and biophysical techniques, including enzymatic assays, CD analyses, and thermal stability studies, we provide novel insights into the impact of the p.T236N mutation on CBS protein function and stability. Our findings shed light on the unique molecular characteristics of this mutation and its potential role in the pathogenesis of CBS deficiency. This study fills a critical gap in the existing literature and lays the foundation for further research aimed at understanding the underlying mechanisms of CBS-related disorders and developing targeted therapeutic strategies for affected individuals.

The CBS^T236N^ mutant displays significant deviations from the CBS^WT^ enzyme. Specifically, CBS^T236N^ exhibits reduced stability and enzymatic activity when compared to the native enzyme. The canonical B-type heme present in CBS^WT^, bound axially to the thiolate group of C52 and the imidazole part of residue H65, serves as a redox detector capable of interacting with external ligands such as CO and NO [30]. This interaction plays a role in regulating CBS activity, ensuring structural integrity, and promoting proper protein folding [31]. The interaction between heme and the PLP active site is believed to occur through molecular interactions involving amino acid residues 258–272 situated at both ends of an α-helix. This helix has been referred to as either helix-α8 or helix-α9 [32,33]. Specifically, at one end of this helix, residue R266 forms electrostatic interactions with the C52 thiolate, while at the opposite end, T257 and T260 engage in a hydrogen bond network with the PLP phosphate group. Functional and spectroscopic studies on clinically relevant variants of R266, T257, and T260 have confirmed the role of these residues in transmitting information regarding the heme’s redox state and ligand binding to the PLP site [32]. We found that the pathogenic mutant in which the side chain of T236 is substituted for a larger side chain of aspargine residue exhibited a significant decrease in overall protein stability and enzyme activity. In addition, since asparagine has a larger side chain compared to threonine due to its amide group, this potentially leads to steric hindrance or altered interactions within the protein structure [34].

The functional diversity of CBS mutations located within the catalytic domain has been studied, revealing that different mutations target various residues with distinct consequences. For instance, mutations affecting residues involved in pyridoxal-5′-phosphate (PLP) binding or catalytic activity may result in a broader spectrum of biochemical phenotypes, including variable responsiveness to pyridoxine supplementation [23,35]. In contrast, the CBS^T236N^ mutation specifically affects residue 236 within the CBS protein’s catalytic domain and exhibits a consistent pyridoxine non-responsive phenotype, suggesting a unique mechanism of pathogenicity. Biophysical characterization studies have demonstrated distinct structural and stability profiles among CBS mutations in the catalytic domain, with some variants exhibiting severe protein destabilization or misfolding [36]. Future comparative studies exploring the functional consequences and structural alterations associated with different CBS mutations within the catalytic domain are warranted to elucidate their diverse pathogenic mechanisms and improve personalized treatment strategies for individuals affected by CBS-related disorders.

Currently, we are exploring the potential of cell culture-based approaches to rescue the mutant CBS protein and restore its enzymatic activity. By utilizing cell culture models expressing the CBS^T236N^ mutant protein, we aim to investigate the efficacy of various pharmacological agents or molecular chaperones in mitigating the functional consequences of the mutation. Our findings provide valuable insights into the feasibility of therapeutic strategies targeting CBS deficiency at the cellular level. While further optimization and validation are necessary, our cell culture trials pave the way for the development of novel treatment modalities aimed at restoring CBS activity and ameliorating the clinical manifestations associated with CBS-related disorders. Additionally, these cell culture-based experiments serve as a valuable platform for screening potential therapeutic candidates and elucidating the underlying molecular mechanisms involved in CBS deficiency pathogenesis. Further studies utilizing these models may facilitate the identification of promising therapeutic targets and inform the development of personalized treatment approaches for individuals affected by CBS deficiency.

## 4. Conclusions

In conclusion, despite extensive research on the CBS protein, the precise molecular mechanisms underlying the severe phenotypes associated with the CBST236N mutant remain elusive. This ambiguity primarily stems from a limited understanding of how this mutation impacts the molecular characteristics of the protein. Therefore, our study aimed to investigate the effects of the p.T236N missense mutation on the biochemical and biophysical properties of CBS. Our findings demonstrate that the p.T236N mutation markedly disrupts the stability of the human CBS protein, resulting in a significant reduction in specific enzyme activity, which correlates with the severe phenotype observed in homozygous patients. Additionally, other potential mechanisms, such as the disturbance of critical protein–protein or protein–cofactor interactions, may also contribute to the observed effects. Through this concerted effort, we aimed not only to deepen our understanding on disease pathogenesis, but also pave the way for more personalized therapeutic interventions tailored to the specific molecular signatures of individual patients. By elucidating the molecular basis of pyridoxine non-responsiveness in CBS deficiency, our research seeks to shed light on a critical aspect of this complex disorder and provide hope for improved clinical management and outcomes for affected individuals.

## Figures and Tables

**Table 1:** Estimated secondary structure content (%) from experimental far-UV CD measurements calculated using the BESTSEL online server [26].

Protein	Helix	Antiparallel	Parallel	Turn	Other
CBS^WT^	15.8	19.9	6.1	12.8	45.3
CBS^T236N^ (25 °C)	15.4	12.9	14.3	14.0	43.4
CBS^T236N^ (90 °C)	5.0	17.3	16.8	13.1	47.6

**Table 2:** Thermodynamic stability parameters for CBS^WT^ and CBS^T236N^, as derived by chemical denaturation experiments at 25 °C.

Protein	ΔG_N-U_ (kcal/mol)	m (kcal mol^−1^ M^−1^)	[D]^50%^ (M)
CBS^WT^	2.41 ± 0.24	−0.76 ± 0.07	3.17 ± 0.12
CBS^T236N^	1.15 ± 0.04	−0.74 ± 0.06	1.55 ± 0.05

## Data Availability

Data are contained within the article.
